# Detection dog efficacy for collecting faecal samples from the critically endangered Cross River gorilla (*Gorilla gorilla diehli*) for genetic censusing

**DOI:** 10.1098/rsos.140423

**Published:** 2015-02-25

**Authors:** Mimi Arandjelovic, Richard A. Bergl, Romanus Ikfuingei, Christopher Jameson, Megan Parker, Linda Vigilant

**Affiliations:** 1 Department of Primatology, Max Planck Institute for Evolutionary Anthropology, Deutscher Platz 6, Leipzig 04103, Germany; 2 North Carolina Zoo, 4401 Zoo Parkway, Asheboro, NC 27205, USA; 3 Wildlife Conservation Society, 2300 Southern Boulevard, Bronx, NY 10460, USA; 4 Working Dogs for Conservation, 52 Eustis Road, Three Forks, MT 59752, USA

**Keywords:** apes, primates, microsatellite, genotyping, canine, survey

## Abstract

Population estimates using genetic capture–recapture methods from non-invasively collected wildlife samples are more accurate and precise than those obtained from traditional methods when detection and resampling rates are high. Recently, detection dogs have been increasingly used to find elusive species and their by-products. Here we compared the effectiveness of dog- and human-directed searches for Cross River gorilla (*Gorilla gorilla diehli*) faeces at two sites. The critically endangered Cross River gorilla inhabits a region of high biodiversity and endemism on the border between Nigeria and Cameroon. The rugged highland terrain and their cryptic behaviour make them difficult to study and a precise population size for the subspecies is still lacking. Dog-directed surveys located more fresh faeces with less bias than human-directed survey teams. This produced a more reliable population estimate, although of modest precision given the small scale of this pilot study. Unfortunately, the considerable costs associated with use of the United States-based detection dog teams make the use of these teams financially unfeasible for a larger, more comprehensive survey. To realize the full potential of dog-directed surveys and increase cost-effectiveness, we recommend basing dog-detection teams in the countries where they will operate and expanding the targets the dogs are trained to detect.

## 2. Introduction

Population estimates derived from genetic capture–recapture studies can be more accurate and precise than traditional ape surveys based on nest counts [1–4]. In addition to population estimates, genetic capture–recapture studies may permit long-term population monitoring by revealing ranging patterns and group composition [1,2,5]. However, the accuracy of mark–recapture and population monitoring studies depend entirely upon effective sampling of the surveyed area. To be effective, the number of samples collected should be at least two-and-a-half times the number of individuals suspected to be in the population [1,6,7]. Furthermore, because the entire study area cannot be searched in one day, subdivision of the landscape into daily search areas is necessary. The search effort expended in these sub-areas should be consistent and each zone should be revisited as many times as possible. In order to collect sufficient samples for genetic mark–recapture analysis, researchers have used specially trained dogs to detect faeces from a variety of target species over large geographical areas (e.g. bears: [8,9] wolves: [10], wolves, pumas, jaguars, anteaters and armadillos: [11]). These dogs can detect scat samples from specific species with a high degree of accuracy and at rates 5–15 times higher than humans [12]. The use of detection dogs should result in increased sample detection rates and accordingly larger sample sizes, therefore allowing for higher levels of precision than standard sample collection regimes. In addition to detecting more samples, it is possible that dogs detect samples in a less biased way than humans. In the case of gorillas, human-directed detection teams tend to be biased towards collecting samples from nest groups as they are easier to detect then single faecal remains; similarly, collection tends to be biased towards large adult faecal samples rather than smaller infant and juvenile samples. Dogs may be able to overcome these biases and provide a more homogeneous capture rate, and therefore, more precise population estimate, than currently possible from human-directed surveys.

The Cross River gorilla (*Gorilla gorilla diehli*) is the most endangered of all the African apes and one of the world's most critically endangered primates [13]. These gorillas are found only in remote and mountainous regions along the Nigeria–Cameroon border and are restricted to approximately 11 localities in areas of forest that range from small fragments of 20 km^2^ to large blocks of over 1000 km^2^ [14]. Estimates based on nest counts suggest the population numbers only 200–300 individuals [14]. This population is Critically Endangered [15] and is under intense threat from bush meat hunting, habitat loss and habitat fragmentation [14]. As a flagship species for the region, their conservation is also critical for the protection of the biodiversity hotspot which they inhabit and the other species that share their habitat.

While recent research has offered a number of insights into the biology of the Cross River gorilla (e.g. [16–20]), several questions critical to their effective conservation remain unanswered, including a precise estimate of the gorillas' current population size [14]. Without this knowledge it is impossible to effectively assess subpopulation viability, prioritize interventions or measure success of conservation activities over time. Despite substantial effort over many years (e.g. [21]), efforts to unambiguously determine population size have been thwarted by the total size of the landscape inhabited by the gorillas (over 2000 km^2^), the difficult terrain, and the reclusiveness of the gorillas. As a result, no DNA-based estimate of population size has been possible and current estimates are based entirely on nest counts, which are prone to significant error [22]. Another important question to address is the degree of connectivity between gorilla localities. Although analysis of non-invasively collected DNA has offered a number of insights into the population structure, genetic diversity and migration between fragmented habitat sites of the Cross River gorillas [16,23], these findings have been limited in their generalizability by small sample sizes from some sites, and a complete lack of samples from others.

Prior to our study, the use of detection dogs for locating faeces had not been attempted in an African tropical forest, an environment where the detection of samples by humans or dogs can be particularly challenging. Here we present the results of a study piloting the use of dogs in the collection of gorilla faeces for genetic analysis to determine whether this approach is suitable for a population-wide study. Our specific goals were to estimate the number of gorillas at two Cameroonian Cross River sites (the smaller and better-studied Kagwene Gorilla Sanctuary and the more expansive and mountainous Mone River Forest Reserve), estimate grouping patterns of the gorillas, and compare the newly identified individual genotypes with those collected from multiple localities 10 years earlier [16] to check for resampled individuals or any individual movement. We also compare the efficiency with which human and dog-directed teams collected samples and assess the relationship between the number of samples and the number of individuals found and the resultant effect on the population estimate, by the two team types. Finally, we draw some conclusions about the applicability of dog-directed faeces detection in mountainous tropical rainforest environments.

## 3. Material and methods

### 3.1 Training of detection dogs

Cross River gorilla faeces collected by field staff in Cameroon and from captive western lowland gorillas (*Gorilla gorilla gorilla*) were initially used to train three Working Dogs for Conservation (WDC) dogs to identify gorilla faeces using methods previously applied in wildlife research [8]. The dogs were trained using a series of increasingly complex detection scenarios, using procedures developed by WDC for the introduction of a new scent target [8,9]. In December 2011, the dogs and their handlers travelled to Cameroon to complete their training via simulated Cross River gorilla searches conducted over a 4 day acclimatization period in the town of Limbé using additional fresh Cross River gorilla faecal samples. Following the acclimatization period, the dog teams travelled to Kagwene Gorilla Sanctuary where final field training was conducted.

### 3.2 Detection dog-directed field surveys

**Figure 1.**
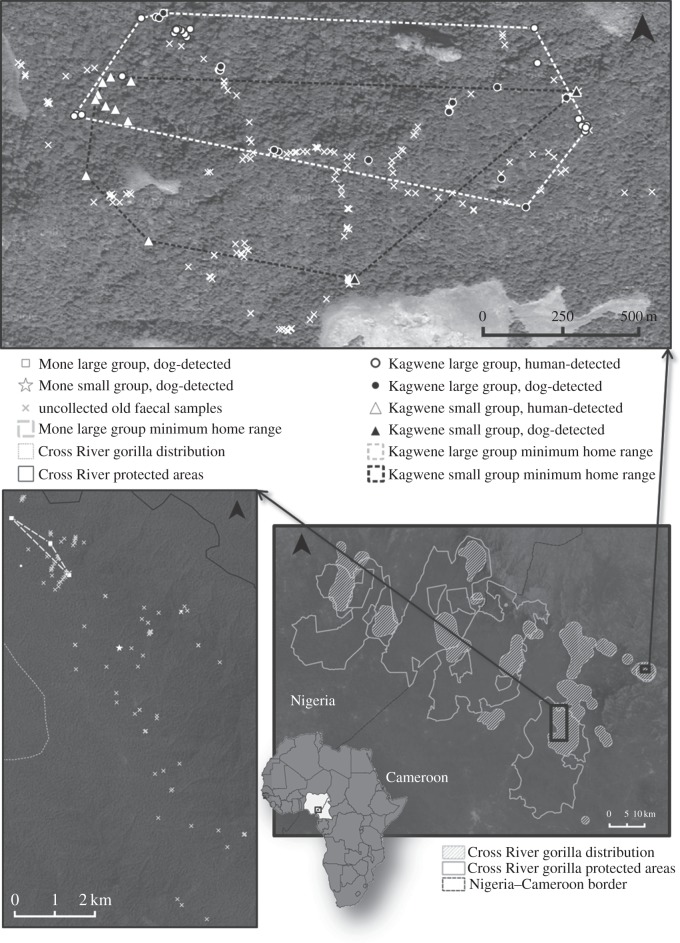
Map of Cross River gorilla sampling locations and ranging. Map of Africa with Cameroon and Nigeria highlighted in white. Bottom, right inset: Cross River region. Solid white lines denote Cross River protected areas, white hatched lines indicate the current range of the Cross River gorilla. Black boxes indicate the sampling area in Mone River Forest Reserve (left) and Kagwene Gorilla Sanctuary (right). Top inset: samples collected from Kagwene Gorilla Sanctuary: circles and triangles indicate samples from the larger and smaller Kagwene groups, respectively. White symbols represent human-located samples, black symbols represent dog-located samples. Minimum home ranges are delineated with dotted lines for the two identified groups (white line for the large group, black line for the small group). Bottom, left inset: samples collected from Mone River Forest Reserve: white circles indicate samples attributed to the larger Mone group, the white star represents samples attributed to the smaller Mone group. Minimum home range of the larger group delineated with a dashed white line. White crosses in both maps indicate old faecal samples identified by the dog-detection team that were not collected for analyses.

Dog-directed searches were conducted in December 2011 and January 2012 at two sites known to be occupied by gorillas: Kagwene Gorilla Sanctuary (19 days) and the northern portion of Mone River Forest Reserve (25 days) (figure 1). Kagwene was selected as the first test site because it is small (19 km^2^), the ranging behaviour of the estimated 20–25 gorillas there is regularly monitored, and the Wildlife Conservation Society (WCS) has a permanent presence at the site. Mone was chosen as the second site as it is more representative of a typical Cross River gorilla locality with a larger forest area (460 km^2^, of which *ca* 100 km^2^ is occupied by gorillas [14]), steep terrain and limited knowledge of gorilla numbers and ranging patterns. Initially, a 1.5 km^2^ grid was used to define sampling units which included the estimated range of the gorillas at each site. Survey teams planned to conduct 1.5 km guided reconnaissance walks [22] across each cell, following a bearing from the edge of the cell towards its centre. This approach was adjusted in the field based on logistical constraints (see Results) and a method that attempted to maximize the number of grid cells explored each day was used. Whenever a dog detected a scat, the team followed the dog to the source of the scent. Gorillas routinely construct nests of vegetation each night, and samples were collected at nest sites or on trails. Two field teams operated concurrently, each consisting of a dog, a dog handler, a field assistant and a guide. Faecal samples for genetic analysis were collected using the two-step ethanol-silica procedure [24], stored in the field for two months and at 4°C thereafter. GPS coordinates of samples and survey routes were recorded using a Garmin GPSmap 62CX receiver.

### 3.3 Human-directed field surveys and comparison with dog-directed surveys

Human-directed searches for faecal samples were conducted at Kagwene for 17 days in December 2011. A grid-based survey was not employed and teams consisting of trackers and field assistants searched areas based on individual knowledge of historical gorilla ranging patterns, and predictions of where gorilla foods would be available. When signs of gorilla presence were observed (e.g. feeding signs, signs of passage), teams would attempt to follow the signs to a nest site to obtain samples. GPS coordinates of samples were recorded using a Garmin GPSmap 62CX receiver. The probability of dogs and humans detecting samples found in nest groups versus those found alone on a trail were compared using a Fisher's exact test.

Results from the 2011 human-directed field surveys may be unrepresentative as the samples were collected from the Kagwene gorilla groups that are under study and followed regularly. It was not possible to conduct human-directed surveys in Mone during the 2012 season. Therefore, sample detection rates from dog-directed searches were compared with sample collection rates of human-directed searches at Mone and Kagwene conducted previously between 2002 and 2004 [16]. Both human-directed and dog-directed surveys were conducted during the dry season to maximize the chance of finding usable samples (i.e. those not destroyed by rain) and to control for environmental differences between the two search methods. Sample detection rate was calculated as the number of fresh samples collected per team day (i.e. two teams collecting samples on the same day is counted as two team days). Only faeces that were 1–3 days old, as determined by field staff with years of experience, were collected as older samples tend not to yield usable genotype results [25]. An approximation of gorilla age was not estimated based on faecal bolus size.

### 3.4 Microsatellite genotyping and analysis

DNA was extracted from faecal samples approximately five months after collection using the QIAmp Stool kit (QIAGEN) with slight modifications [24]. DNA quantification was performed using a 5^′^-nuclease assay targeting a highly conserved 81 bp portion of the c-myc proto-oncogene [26].

At least three independent amplifications from each DNA extract along with a minimum of three negative controls were performed at 13 microsatellite loci using a two-step multiplex polymerase chain reaction (PCR) method [27] with the following modifications: (i) in the initial multiplexing step, 16 loci [27] were multiplexed using the Type-it Microsatellite PCR kit (Qiagen) in 20 μl reaction volumes (including 5 μl template DNA); (ii) PCR thermocycling was performed in a PTC-200 thermocycler (MJ Research) with the following parameters: initial denaturation for 5 min at 95°C, 30 cycles of 20 s at 94°C, 90 s at 57°C and 30 s at 72°C, and a final extension of 30 min at 72°C; and (iii) primers were also multiplexed in the second step using the Type-it Microsatellite PCR kit (Qiagen) in 10 μl reaction volumes (including 2.5 μl of 1.5 : 100 diluted 1st-step multiplex PCR product) in the following combinations: (d10s1432 (0.5 μM)-d14s306(0.5 μM)-d5s1457(0.1 μM); d5s1470(0.6 μM)-d4s1627(0.1 μM)-d2s1326(0.2 μM); vWF(0.4 μM)-d7s2204(0.6 μM)-d16s2624(0.1 μM); d7s817(0.2 μM)-d1s550(0.2 μM)-d8s 1106(0.2 μM)-d6s1056(0.2 μM)). Second-step thermocycling conditions were as above except primer-combination-specific annealing temperatures were used (varying from 55°C to 60°C) and the annealing step lasted 3 min. The sex of each individual was determined by amplifying a segment of the *x*–*y* homologous amelogenin gene in a one-step PCR [28].

PCR products were electrophoresed on an ABI PRISM 3100 Genetic Analyser and alleles were sized relative to an internal size standard (ROX labelled HD400) using GeneMapper software v. 3.7 (Applied Biosystems). Heterozygous genotypes were confirmed by observing each allele twice in two or more independent reactions. Apparent homozygous genotypes were confirmed by observing only a single allele in at least three to five independent observations depending on DNA quantity [27].

### 3.5 Discrimination of individuals

We used Cervus v. 3.0 to identify samples with matching genotypes. We determined the minimum number of loci necessary to obtain PID_sibs_≤0.001 to ensure with 99.9% confidence that two matching samples originated from the same individual [29]. Consensus names and genotypes were attributed to matching samples. The consensus genotype was used in all subsequent analyses. Genotypes from different samples mismatching at three or fewer loci were re-examined for possible genotyping errors and additional genotyping was undertaken to resolve any ambiguities.

### 3.6 Gorilla group determination

Individual genotypes derived from samples collected by dog- and human-directed searches were pooled and the number and minimum composition of gorilla groups at Mone and Kagwene were estimated using three possible schemes. First, we assumed that samples from individuals collected together on the same day at the same GPS location (same nest site or multiple fresh faecal remains found together) belong to individuals from the same group (grouping scheme 1). Second, because unique GPS coordinates were taken for most samples even when collected as close as 1 m apart, samples were assumed to come from the same group if they met the following criteria: (i) they were found on the same day, (ii) they were judged as equally old by the field team, and (iii) they were considered by the field team to belong to a single nest group (grouping scheme 2). Third, based on the fact that nest sites contained samples up to 54 m apart (±3 m GPS estimated position error), samples were considered to come from the same group if: (i) they were found on the same day, (ii) they were judged as equally old by the field team, and (iii) they were found within 57 m of each other (grouping scheme 3). Individuals were then further linked under the assumption that if individuals A and B were found at sampling event one and individuals A and C were found at sampling event two then individuals A, B and C are all part of one group [1]. Therefore, group attribution could not be assigned to individuals whose faeces were sampled only once and not together with faeces of other individuals. Minimum home range was determined in QGIS v. 2.2.0-Valmiera through the creation of minimum convex polygons using the GPS locations of individuals based on pooled samples under grouping scheme 3 [1].

### 3.7 Population estimation by genetic analysis

We calculated genetic capture–recapture estimates using the maximum-likelihood two innate rates model (ML-TIRM) estimator implemented in the software Capwire (www.cnr.uidaho.edu/lecg; [6]). In a previous study on western lowland gorillas it was found that the ML-TIRM estimator is the most reliable of the available published estimators, while other methods appear to underestimate the population size [1]. Furthermore, other studies have shown that although the confidence intervals of the ML-TIRM estimator are larger than those from other estimators, they always capture the true population size within their limits [30]. The approach assumes a closed population (i.e. no births, deaths or migration in the sampling interval) and a recapture probability equal to the capture probability. It also accounts for capture heterogeneity by classifying individuals as having either low or high capture probabilities [6]. As migration of individuals between the Mone and Kagwene areas is not considered probable [16], we calculated population estimates for each locality separately. We grouped each set of samples into a single-sampling session scheme and used consensus names to identify the number of times each individual was captured. Individuals found at the same location on the same day are false recaptures, so only one sample representing that individual at that location was kept in the dataset. Therefore, population estimates were calculated following grouping schemes 1, 2 and 3 as described above (i.e. under grouping scheme 1, only individuals identified more than once at the same GPS location were considered false recaptures, whereas under grouping scheme 3, individuals identified more than once within 57 m of each other on the same day were considered false recaptures). Furthermore, we calculated a population estimate employing all of the Kagwene samples as well as using samples from dog- and human-directed searches separately.

### 3.8 Detection of migrants

Using the same parameters as in the ‘Discrimination of individuals’ section above, we used Cervus v. 3.0 to check for samples from this study with genotypes matching those from a previous study of Cross River gorillas from multiple localities [16]. As that study [16] was able to infer the presence of migrant gorillas between some Cross River gorilla localities, we also wanted to determine if any of our newly identified individuals from Mone and Kagwene were migrants from another Cross River locality. To do so, we ran a Structure v. 2.1 analysis [31] with the original 2007 dataset and original parameters but with the addition of our newly identified individuals from this study.

## 4. Results

### 4.1 Dog- and human-directed surveys

The combination of the rugged and remote terrain and the grid cell sampling strategy optimal for the mark–recapture analysis presented significant challenges to the dog teams. Steep terrain and dense vegetation caused teams to move slowly (dogs were impeded by dense vines but mobility of the dog handlers was generally the limiting factor in all searches with dogs). Travelling from an edge of a 1.5 km^2^ grid cell towards its center and emerging at a different edge, as specified in the original sample collection protocol, proved impossible. The presence of cliffs (not always evident on maps) and the substantial distance between the far side of a grid cell and field camps necessitated revision of the search protocol. By necessity, looping transects were used, in which the dog teams followed a route that returned them close to the start point of their search. In addition, owing to the large size and rugged nature of the study sites, access to grid cells was highly dependent upon camp site location. At Mone, it was possible to search cells contiguous to the one in which the field team was camped. However, this necessitated moving camps in order to reach grid cells. The large size of the field team, substantial amounts of equipment and supplies necessary to support the dogs, and the logistics of moving camps meant that not all grid cells could be searched. These issues, combined with the limited time available, meant that repeated sampling of grid cells was not possible in most cases. To mitigate the impact of these challenges the dog teams focused on searching as many cells as possible, and then prioritizing cells within which fresh faeces was located for re-survey. While this probably increased the absolute number of samples detected it may have contributed to false recaptures and meant that significant areas went unsearched.

Handlers could see dogs well enough to determine detection distances (the distance from the location where the dog exhibited a ‘change of behaviour’ (where the handler can easily see where a dog catches the target scent and begins to work towards the sample) to the location of the sample) for 35% of the collected faeces. At Kagwene, the observed distances ranged from 0 to 60 m, the majority being less than 20 m. At Mone, only twice (10%) were handlers able to observe the detection distance of collected faeces, and both times it was zero, meaning that the dog was upon it before appearing to smell it.

The dog teams expended 44 team days (i.e. a detection dog-directed survey team actively looking for samples) between the two field sites (19 days in Kagwene and 25 days in Mone). Dog teams located 43 fresh (1–3 days old) faecal samples (26 in Kagwene, 17 in Mone). A total of 288 (187 in Kagwene, 101 in Mone) old faecal samples were also located (figure 1). These samples were not collected as they were assumed to be too decayed to be useful for genetic analysis, but provide additional information about the gorillas' ranging behaviour. Each of the three dogs located both fresh and old faeces. The 43 fresh samples located by the teams correspond to an average of 0.97 samples per team day overall. At Kagwene, the fresh sample collection rate was 1.37 samples per team day, while at Mone it was 0.68 samples per team day. In comparison, during a previous human-directed sweep search in Mone & Kagwene [16], 175 team days yielded 75 fresh samples, or 0.43 samples per team day. Human-directed searches never found old faecal samples. Overall, the rate of faeces detection by dogs was significantly higher than by humans (Fisher's exact test, *p*=1.19×10^−12^).

In this study, fresh faecal samples located away from nest sites were more likely to be detected by dogs (*n*=31/43 (72%)) than by humans (*n*=13/33 (39%)) (Fisher's exact test, *p*=0.005). When omitting samples that did not produce viable genotypes (see below) the significance still holds (*n*_dogs_=28/39, *n*_humans_=13/33, Fisher's exact test, *p*=0.008). Even when comparing samples that produced usable genotypes collected in Kagwene under the less conservative assumptions of grouping scheme 3 (that samples found within 57 m belong to the same group) the detection probability of dog-directed searches was higher (*n*_dogs_=10/22, *n*_humans_=6/33, Fisher's exact test, *p*=0.04).

### 4.2 Genotyping results

Of the 76 samples extracted, 72 samples produced usable genotypes, for a total genotyping success of 95%. The four samples that did not yield genotypes were all from dog-directed searches. However, two of these samples were suspected in field of possibly not being of gorilla origin. Of the 72 samples, 63 were amplified successfully at all 13 loci, eight amplified at 11–12 loci and one sample amplified at only eight loci. Total genotyping success was 98.3%.

The PID_sibs_ for all matches between genotyped samples was less than 0.0006 except for one sample which was only genotyped at eight loci (CRC-035). For this sample, the PID_sibs_ match to the consensus genotype (G014) was 0.002, however, we are confident in this match as an additional three unconfirmed alleles were matches and there were no other candidate matches. Consensus genotypes were 99.6% complete. Consensus genotypes mismatched one another at three or more loci, a demonstration that there is very little genotyping error present in the dataset [32].

### 4.3 Grouping inferences

**Figure 2.**
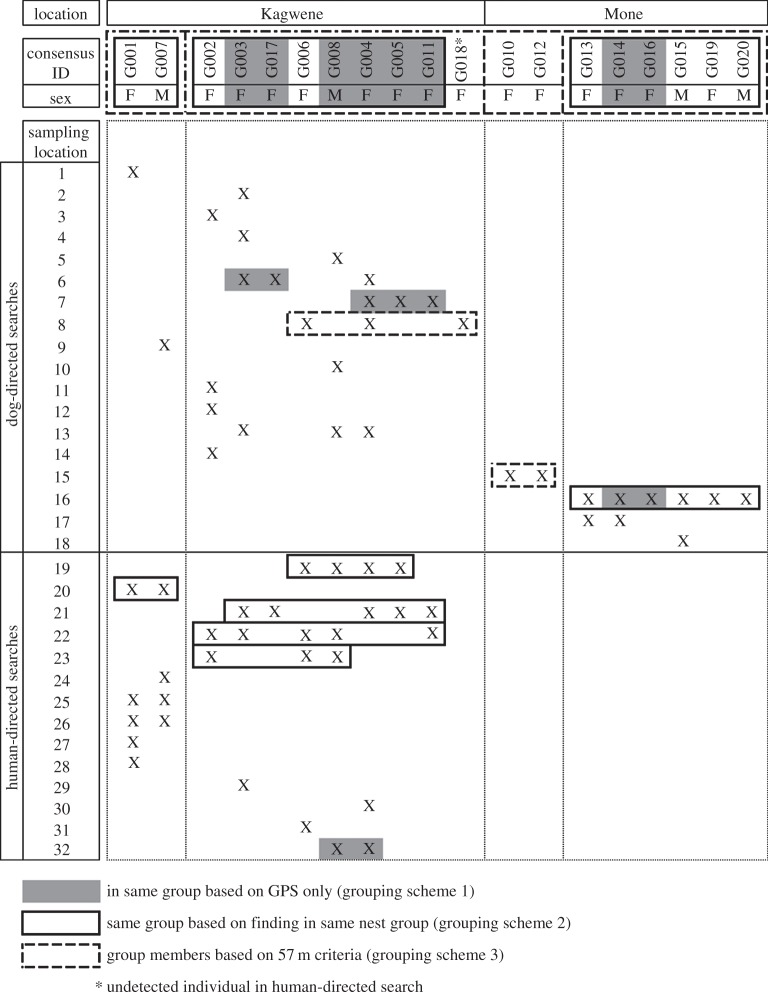
The composition of the Kagwene and Mone Cross River gorilla groups as ascertained by both dog- and human-directed searches and according to grouping scheme. Detections of each gorilla by unique sampling location marked with an ‘X’. M refers to males, F to females. Assessment of group membership according to grouping scheme shown as follows: grey boxes: grouping scheme 1 (samples found at the same GPS location are considered group members); solid-outlined boxes: grouping scheme 2 (samples found in same nest group are considered the same group); dashed-outlined boxes: grouping scheme 3 (samples of the same age, found on the same day and within 57 m of one other are considered the same group).

The 72 successfully genotyped samples comprised 19 individuals, four of which were male while 15 were female. Eleven individuals were identified in Kagwene (two males, nine females), and eight in Mone (two males, seven females). In Kagwene, all individuals were detected in both the dog- and human-directed surveys except G018, a female that was only detected in the dog-directed search (figure 2).

Using pooled samples from the dog-directed and human-directed searches and grouping scheme 3, two groups were identified in Kagwene and two in Mone. Both of the Kagwene groups were sampled multiple times supporting the proposition that two distinct groups are present at the site (WCS 2011, unpublished data). One such group consists at a minimum of one male and a female, while the other contains at least eight females and one male. It is important to note that only the minimum number of group members can be inferred and also that limited sampling may result in the inference of such clusters of gorillas even if they are parts of a single group. In a parentage analysis (data not shown), the two males present in Kagwene constitute a possible father–son pair. The home ranges of the two groups overlapped.

At Mone, two groups were also inferred, however, one group was only detected a single time, only with grouping scheme 3 and is made up of two females, thus strongly suggesting that these two gorillas are actually part of another group. The other group detected at Mone consists of at least four females and two males and all were found in the same nest group. Interestingly, a parentage analysis suggested that the two males were not a father–son pair as there were mismatches between their genotypes at three loci (data not shown); this, however, does not preclude the possibility that these two individuals could be brothers. The minimum home ranges of the two ‘groups’ were spatially distinct and separated by 3 km based on our sampling, although with such limited sampling it is unclear whether these are two parts of the same group or not.

### 4.4 Population estimate

**Figure 3.**
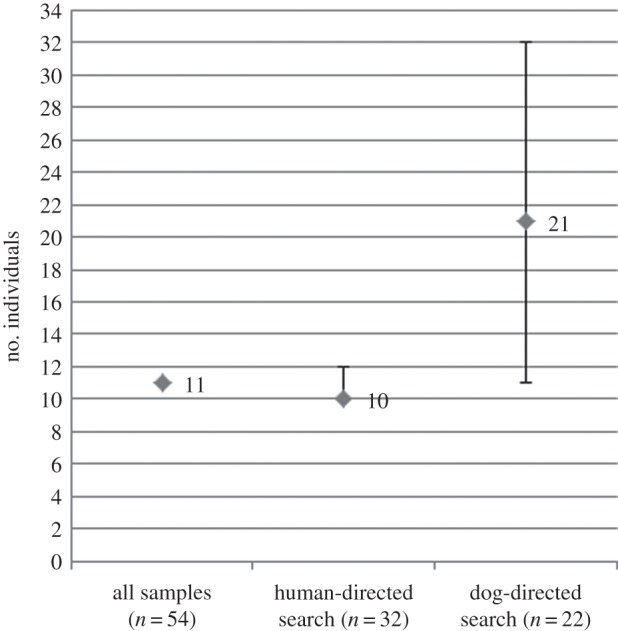
Kagwene population estimate using all genotyped samples, human-directed search samples only, and dog-directed search samples only (results were the same for all three grouping schemes). Error bars represent 95% confidence intervals.

Samples were analysed according to sampling location, sampling method (dog versus human and pooled) and grouping scheme (1, 2 or 3). Recapture counts did not very greatly according to grouping scheme (table 1). Grouping scheme did not alter the population estimate for the Kagwene samples when taken all together or when separated into dog- and human-directed search samples. The recapture rate from the Kagwene Gorilla Sanctuary was very high (10 out of 11 individuals sampled more than once), with several individuals being sampled five or more times. The resampling rate from human-directed searches was higher (90%) than those from the dog-directed searches (36%). When all samples were taken together the population estimate for Kagwene was 11 individuals with no variation in the estimate (figure 3). For the human-directed search the estimate was 10–12 individuals with a point estimate of 10, while for the dog-directed search the estimate was 21 but ranging from 11 to 32 individuals.

**Table 1.** Recapture counts and rates (proportion of individuals sampled more than once) for the three grouping schemes when samples are pooled or when dog-directed and human-directed search samples are evaluated separately. (*a*) grouping scheme 1: only matching samples found at the same GPS locations are considered false recaptures (and one sample is omitted from the dataset); (*b*) grouping scheme 2: samples found in same nest group are considered the same group, therefore, matching samples found in the same nest group are considered false recaptures; and (*c*) grouping scheme 3: samples found within 57 m of one other on the same day are considered the same group, therefore, matching samples found using this grouping criteria are considered false recaptures.

	no. individuals in capture category			
	Mone	Kagwene		
no. captures	(dogs)	dogs	humans	all
(*a*) grouping scheme 1				
1	2	7	1	1
2	3	0	3	1
3	3	1	1	2
4	—	3	4	0
5	—	—	0	2
6	—	—	1	1
7	—	—	—	3
8	—	—	—	1
recapture rate:	75%	36%	90%	91%
(*b*) grouping scheme 2				
1	3	7	1	1
2	4	0	3	1
3	1	1	1	2
4	—	3	4	0
5	—	—	0	2
6	—	—	1	1
7	—	—	—	3
8	—	—	—	1
recapture rate:	63%	36%	90%	91%
(*c*) grouping scheme 3				
1	4	7	1	1
2	4	0	3	1
3	—	1	1	2
4	—	3	4	0
5	—	—	1	2
6	—	—	—	2
7	—	—	—	2
8	—	—	—	1
recapture rate:	50%	36%	90%	91%

**Figure 4.**
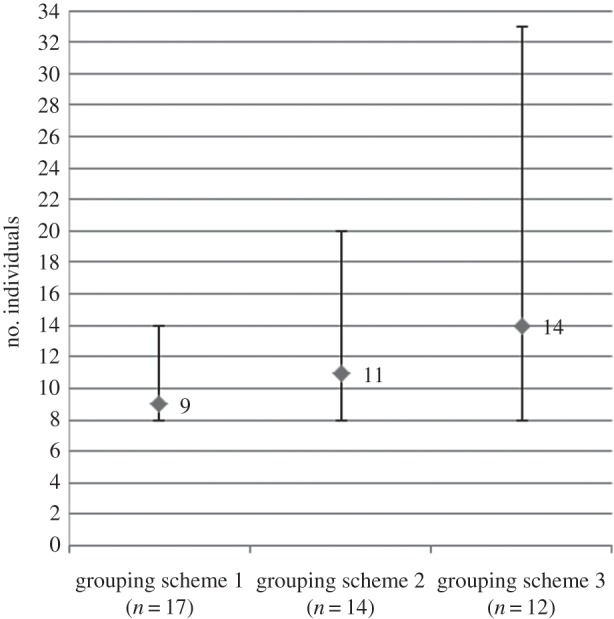
Mone population estimate according to which grouping scheme was applied (see table 1 for details). Error bars represent 95% confidence intervals.

Grouping scheme did, however, alter the results obtained for the Mone estimate with both estimate and confidence interval width increasing according to the grouping scheme (table 1). Depending on the grouping scheme used, we estimated that between 9 and 14 individuals were present in Mone, with an approximate 95% confidence interval of 8–33 individuals (figure 4).

### 4.5 Detection of migrants and gene flow

By comparing the genotypes generated in this study with those produced using samples collected some 10 years previously, we found that of the 11 individuals identified from Kagwene, nine had been previously identified by Bergl & Vigilant [16] and of the eight identified at Mone, three had been previously identified (PID_sibs_<0.001). We found no suggestion that the two newly identified Kagwene gorillas (G017, G018), and five Mone gorillas (G010, G012, G014, G015, G020) derived from a different locality than where they were found.

## 5. Discussion

Although our field study was limited by time and financial constraints, we demonstrate here that dog-directed searches for ape faeces for the purpose of genetic analyses, hold much promise provided some limitations to their implementation are overcome. In only two weeks at each Cross River locality, we were able to obtain accurate population estimates at both sites when using dog-directed searches, albeit with low precision. The low precision of the estimates is a function of the limited time the dogs could spend in the field but this can be offset as described below. We showed that dog-directed searches lead to improved population estimates by overcoming several of the limitations of human-directed searches including increased detection of solitary faecal piles and decreased bias towards collecting certain individuals.

### 5.1 Population estimate and group composition

We obtained a population estimate of 10–12 individuals at Kagwene using the human-detected samples and between 11 and 32 individuals using the dog-detected samples. Additionally, we estimate between 8 and 33 individuals are present at Mone. Ongoing monitoring work at Kagwene has identified at least 18 individuals via observation and previous genetic work has identified a minimum of 15 different animals [16]. Furthermore, previous surveys suggest the presence of between 20 and 25 individuals at Kagwene and 20–30 individuals at Mone [14]. Interestingly, despite the large confidence intervals, grouping schemes 2 and 3 as used with the dog-directed search samples gave estimates closest to the suspected number of individuals at the sites. Furthermore, the true population size should fall within the confidence interval of this estimate according to previous simulation work [30]. Therefore, the human-directed search seems to grossly underestimate the number of individuals, probably owing to bias towards recapturing particular individuals and creating a false rate of recapture. This is reinforced by the observation that despite a larger number of detected samples, the human-directed search detected one fewer individual than the dog-directed search. Furthermore, as can be seen in the variation of sampling scheme with the Mone dog-directed search samples, as the sampling scheme becomes more stringent, and the number of potential false recaptures are removed from the dataset, the population estimate increases, again suggesting that the human sampling is heavily biased and in violation of the mark-recapture estimator assumptions. Some of the human sampling bias exhibited here might be decreased by implementing a more grid-based system so that the dog- and human-directed searches could be more comparable. However, human searches generally rely on finding gorilla trails and then following them backwards to the precious night's nest as otherwise detection rates are very low, even when a grid-based system is used [4,33]. Taking this into consideration, the bias we found appears to be more characteristic of the species leading the searches than the method of searching. The large confidence interval surrounding the dog-directed estimates, especially with such small population sizes, currently makes monitoring the Cross River gorilla population impractical. However, with additional sampling and an increased frequency of true resampling events, the confidence interval would decrease significantly.

The data from Mone River Forest Reserve produced a low precision population estimate of 8–33 individuals. While this accords with previous estimates of the size of the population at Mone, the lack of precision means that our knowledge of the estimated number of gorillas has not improved. The imprecision of the scheme 3 Mone estimate is in part owing to the low rate of recapture (only 50% of the individuals were recaptured, and each of these were recaptured only once). Additionally, the large area occupied by gorillas in northern Mone meant that it is highly likely that other gorilla groups were missed by the survey teams. Logistic and time constraints meant that only a limited portion of the gorillas' estimated range was covered by the survey and most areas could not be surveyed repeatedly. It should be noted however, that this pilot survey took place over only 25 days, suggesting that with increased effort and more resampling bouts, more precise estimates could be obtained.

The human-directed sampling in Kagwene provided slightly better group resolution owing to the larger number of samples and tendency to detect nest groups (and therefore more samples were linkable). However, the dog-directed sampling identified one additional member of the larger group and population. At Kagwene, there are probably at least two distinct groups. As the two males are a possible father–son pair this suggests that the smaller group could represent a new silverback (the son) with a new small group or an old silverback (the father) with a remaining female. At Mone there may be two groups and if so, then at least one group is larger than detected here as we did not detect a male in the smaller group and western gorilla females are not known to range without a male [34]. In the large Mone group, the two males are not a father–son pair. It is possible that the group silverback was not sampled and these are non-silverback brothers, however, it is rare to not find silverback faeces at a nest site [3]. Neither of these males were detected in a previous genetic sampling of the area [16].

### 5.2 Dog-directed surveys

Dog-directed searches were successful at locating fresh gorilla faecal samples at a rate higher than with human-directed surveys on a catch per unit effort basis (i.e. samples found per team day). The detection dog-directed surveys were an effective method for locating usable samples and also provided a less biased sampling than those from human-directed searches, resulting in more individuals detected and a more reliable population estimate. However, several factors constrain the broader application of this approach. First, the cost of using detection dogs, at least as applied in the current project, was significant when compared with more traditional methods. Total costs for the project were over $98 000 USD. When just field expenses are considered (i.e. no PI or other supervisory salaries, office costs, indirect costs, etc.), the dog-detected samples cost $1479 USD per sample to collect. Even though human-directed searches returned fewer samples, the cost per sample was only $224 USD. Although the time required would be significantly greater than for the dog-directed surveys, one could theoretically collect seven times as many samples using human-directed searches for the same cost.

The logistical challenges associated with using detection dog teams based in the USA were also considerable. Both dogs and handlers required time to acclimatize to environmental conditions in Cameroon. Additionally, although the dogs remained healthy throughout the project, there was considerable concern that they would be susceptible to potentially fatal diseases (e.g. onchocerciasis). These challenges, combined with difficulties inherent to the sampling regime, remote and poorly mapped field sites, rugged terrain and the low density of the gorillas mean that a population-wide survey using United States detection dogs would take many months and cost several hundred thousand dollars.

The current project, despite the challenges we encountered, did provide valuable insights into how to best maximize efficiency in using scat-detecting dogs for conducting conservation activities. In particular, we recommend the following.

#### 5.2.1 Establishment of a regional detection dog program

The majority of the costs associated with this project were for handler salaries and weekly contract fees for the handlers and use of the dogs, so even if this pilot study had been extended in time, costs would have continued to significantly increase. A detection dog programme based in Central or West Africa and using local dogs (which are less susceptible to disease) and handlers would be considerably less expensive than using expatriate handlers and internationally based dogs. Such a programme could be a resource for multiple projects and organizations and several agencies have expressed interest in partnering on developing such an initiative. A detection dog programme focused on customs enforcement (but without a field detection component) has recently been established in Gabon (http://www.wagtailuk.com/gabons-sniffing-detectives/) and detection dogs are used in an anti-poaching context in Rwanda's Virunga National Park (http://virunga.org/projects/congohounds/).

#### 5.2.2 When using detection dogs, broaden the scope of the questions to be answered and the study species

Detection dogs are capable of searching for faeces from multiple species simultaneously [11]. Dogs should be trained to locate samples from multiple target species.

#### 5.2.3 Focus on lowland sites

Perhaps the main challenge faced by the detection dog teams was the rugged terrain of the Cross River area. Even though dogs can cover large areas quickly and easily tackle rough terrain, they will always be constrained by their handlers who are necessary for collecting the samples. If the dogs' considerable detection abilities were employed in locations where the terrain was not such an obstacle for handlers, they would probably perform much better than they did in the current project. Projects located in flat/lowland areas would benefit considerably from the use of detection dogs and could employ the grid-based sampling regime we originally intended to use.

#### 5.2.4 Use detection dogs for projects that can use older faecal samples or other evidence of animal presence

In this study, the detection dogs located almost 300 faecal samples that were definitively from gorillas, but which probably were too old for use in genetic analysis. Such samples still represent a valuable source of information on a species' range, and if dogs were trained to identify multiple target species one could rapidly accumulate accurate ranging data that would be difficult to acquire in other ways. In the context of Cross River gorilla conservation, detection dogs may still be useful for better documenting the range of the gorillas, which remains poorly understood.

## Data accessibility

Genotype data available from the Dryad Digital Repository: http://dx.doi.org/10.5061/dryad.st61k.
